# VP2 of Infectious Bursal Disease Virus Induces Apoptosis via Triggering Oral Cancer Overexpressed 1 (ORAOV1) Protein Degradation

**DOI:** 10.3389/fmicb.2017.01351

**Published:** 2017-07-19

**Authors:** Yao Qin, Zhichao Xu, Yongqiang Wang, Xiaoqi Li, Hong Cao, Shijun J. Zheng

**Affiliations:** ^1^State Key Laboratory of Agrobiotechnology, China Agricultural University Beijing, China; ^2^Key Laboratory of Animal Epidemiology and Zoonosis, Ministry of Agriculture, China Agricultural University Beijing, China; ^3^College of Veterinary Medicine, China Agricultural University Beijing, China

**Keywords:** infectious bursal disease virus (IBDV), oral cancer overexpressed 1 (ORAOV1), VP2, apoptosis, degradation

## Abstract

Infectious bursal disease (IBD) is an acute, highly contagious and immunosuppressive avian disease caused by IBD virus (IBDV). Cell apoptosis triggered by IBDV contributes to the dysfunction of immune system in host. VP2 of IBDV is known to induce cell death but the underlying mechanism remains unclear. Here we demonstrate that VP2 interacts with the oral cancer overexpressed 1 (ORAOV1), a potential oncoprotein. Infection by IBDV or ectopic expression of VP2 causes a reduction of cellular ORAOV1 and induction of apoptosis, so does knockdown of ORAOV1. In contrast, over-expression of ORAOV1 leads to the inhibition of VP2- or IBDV-induced apoptosis, accompanied with the decreased viral release (*p* < 0.05). Thus, VP2-induced apoptosis during IBDV infection is mediated by interacting with and reducing ORAOV1, a protein that appears to act as an antiapoptotic molecule and restricts viral release early during IBDV infection.

## Introduction

Infectious bursal disease virus (IBDV) attacks the bursa of Fabricius (BF), triggering massive destructions of developing B-lymphocytes (Sharma et al., 2000; Liu and Vakharia, 2004) and impeding the immunological maturation (Biro et al., 2011; Liang et al., 2015). The damaged immune system inflicts severe immune-suppression on the infected chickens, accompanied with increased susceptibility to other infectious diseases (Schat and Skinner, 2013). IBDV infection can cause a high mortality in young chickens of 3–6 weeks when they are at the maximal stage of BF development (Mahgoub, 2012). As a causative agent of a highly contagious disease breaking down the host defense system, IBDV leads to great economic losses to the poultry industry across the globe.

Infectious bursal disease (IBD) virus belongs to the Birnaviridae family and the virions are non-enveloped icosahedra enclosing a bipartite double-stranded RNA (segments A and B) (Azad et al., 1985). Segment B encodes VP1, a RNA-dependent RNA polymerase linked to the virus genomic segments, while free VP1 in the particles also have been reported (Muller and Nitschke, 1987; Lombardo et al., 1999). Two partially overlapping open reading frames (ORFs) in Segment A (3.17 kb) encode the major components of the virus (Spies et al., 1989). The first ORF encodes a non-structural protein VP5 (17 kDa), and the other encodes pVP2-VP4-VP3 polyprotein (∼110 kDa) which is then cleaved by the viral protease VP4 (28 kDa) to release pVP2 (512 residues, 54.4 kDa) and VP3 (32 kDa) in the infected cells (Lejal et al., 2000). The C terminus of pVP2 is further processed by both VP4 and the puromycin-sensitive aminopeptidase, then finally cleaved by itself to yield mature VP2 (Irigoyen et al., 2009, 2012). The mature VP2 with VP3, a scaffold protein with RNA binding activity (Saugar et al., 2005; Casanas et al., 2008), assemble the capsid accompanied with some peptides arising from the cleaved pVP2 (Chevalier et al., 2005).

It is well-known that apoptosis is responsible for the rapid depletion of lymphocytes during IBDV infection, which is important for IBDV-induced immune-suppression and its pathogenesis (Vasconcelos and Lam, 1994; Rodríguez-Lecompte et al., 2005; Khatri and Sharma, 2009). Two viral proteins, VP5 and VP2, are involved in the programmed cell death process. Decreased apoptosis was observed in the cells infected with IBDV mutant deficient of VP5 (Yao et al., 1998; Yao and Vakharia, 2001). Our previous studies have shown that VP5 is the major trigger of the IBDV induced apoptosis in DF-1 cells, interacting with voltage-dependent anion channel 2 (VDAC2) and receptor of activated protein kinase C 1 (RACK1) (Li et al., 2012; Lin et al., 2015). Unlike VP5 whose cytotoxicity has been only found in the host cells so far, VP2 protein exhibits apoptotic activity in a variety of cell lines including mammalian cells (Fernandez-Arias et al., 1997; Shin et al., 2014). Experimental evidence demonstrates that VP2 expression, using vaccinia virus or plasmid-based vectors, shuts off cellular protein synthesis followed by cell apoptosis, which can be counteracted by Bcl-2 overexpression and associated with the activation of PKR pathway (Fernandez-Arias et al., 1997; Busnadiego et al., 2012). However, the detailed mechanism underlying VP2-induced apoptosis remains largely unknown.

As a unique component of icosahedral capsids, VP2 determines the virulence and antigenicity of IBDV (Brandt et al., 2001). With the growing concerns of more-virluent IBDV generated by the intensive use of live attenued vaccines, and the interference of maternally derived IBDV-specific antibodies to vaccines (Muller et al., 2012), the development of recombinant DNA-IBDV vaccines expressing VP2 becomes a hot issue. However, the apoptotic activity of VP2 is adverse to the application of the related recombinant vaccines. On the contrary, VP2 induced cell death for cancer treatment since it also acts on human cancer cell lines, showing the potential of anti-tumor application (Shin et al., 2014). Thus, it is of importance to elucidate the mechanism of VP2 induced apoptosis. Here we demonstrate that VP2 interacts with oral cancer overexpressed 1 (ORAOV1), a pivotal regulator of cancer cell growth (Jiang et al., 2010; Li et al., 2015) and reactive oxygen species (ROS) production (Togashi et al., 2014; Zhai et al., 2014). Remarkable reduction of ORAOV1 was observed in cells either with transient expression of VP2 or IBDV infection, accompanied with marked cell apoptosis. Furthermore, overexpression of ORAOV1 inhibited the apoptotic activity of VP2 in Hela cells and IBDV-induced apoptosis in DF-1 cells, supporting the critical role of ORAOV1 in VP2-induced apoptosis.

## Materials and Methods

### Cells and Virus

HeLa, DF-1, and HEK293T cell lines were all obtained from ATCC, grown in Dulbecco’s modified Eagle’s medium (DMEM) (Invitrogen, United States) supplemented with penicillin (100 U/ml), streptomycin (100 mg/ml) and 10% fetal bovine serum (FBS) in the incubator. IBDV *Lx* is a cell culture-adapted strain, kindly provided by Jue Liu (Beijing Academy of Agriculture and Forestry, Beijing, China).

### Reagents, Chemicals and Antibodies

RNAiMAX (Invitrogen, United States) and jetPRIME^TM^ were obtained from Polyplus-transfection Biotechnology Company (France). 4, 6-Diamino-2-pheny- lindole (DAPI), anti-poly (ADP-ribose) polymeras [poly(ADP-ribose)polymerasepoly(ADP-ribose)polymerasePARP] and MitoTrack Green were purchased from Beyotime Biotechnology (Nanjing, China). Endotoxin Free Plasmid Preparation Kits and EASY spin plus RNA extraction kit were purchased from Aidlab (China). pCMV-Myc, pRK5-FLAG, pDsRed-monomer-N1 and pEGFP-N1 plasmid vectors were obtained from Clontech. Anti-GAPDH (CW0100) antibody was obtained from Kangwei Biological Company (Beijing, China). Anti-FLAG M2 (F1804) antibody and Rabbit anti-ORAOV1 polyclonal antibodies (SAB4300898) were purchased from Sigma (United States). Anti-c-Myc (sc-40), anti-GFP (sc-9996) and anti- β-actin (sc-1616-R) antibodies were obtained from Santa Cruz Biotechnology (United States). Anti-IBDV VP2 McAb (Clone ID: EU0205, which specifically recognizes 394 to 410aa of VP2) was purchased from CAEU Company (Beijing, China). Anti-Caspase-3 (9610) was purchased from Cell Signaling Technology. Caspase-3, Caspase-8 and Caspase-9 colorimetric assay kits were obtained from BioVision (United States), and PE Annexin-V apoptosis detection kit was purchased from BD Pharmingen (United States).

### Constructions of Recombinant Plasmids

*vp2* gene containing the first 452 codons was cloned from IBDV strain *Lx* as previously described (Li et al., 2012). Human *ORAOV1* (*hORAOV1*) gene was cloned from the cDNA of Hela cells using specific primers (sense primer: 5′-GGC TGC TTG CCG CTA TGG- 3′, and antisense primer: 5′-TCA CCA CCT CCT CAAA- 3′) (GenBank ID: 220064) and chicken *ORAOV1* (c*ORAOV1*) gene was cloned from the cDNA of chicken spleen with the primers (sense primer: 5′-TGT AGC GGC GCG ATG GCG- 3′, and antisense primer: 5′-CAT CCA TGT TTA CCT TCA- 3′) (GenBank ID: 423142). *ORAOV1* and *vp2* genes were constructed into the indicated plasmids by standard molecular biology techniques. All the primers were synthesized by Sangon Biotechnology (Beijing, China).

### Apoptosis Assay

Hela cells (6.0 × 10^5^) were seeded on six-well plates and cultured for 12 h, followed by transfection with pEGFP-N1 or pEGFP-vp2 plasmids (500 ng per well). Twenty-four or 48 h after transfection, cells were trypsinized and stained with PE Annexin-V alone or double stained with 7AAD and PE Annexin-V using apoptosis detection kit per the manufacturer’s instructions (BD Pharmingen^TM^). The cells were then analyzed by flowcytometry. GFP-positive cells were gated for further analysis of apoptotic cells with CellQuest software (BD). Hela or DF-1 cells were transfected with siRNAs against ORAOV1 or siRNA negative controls for 48 h. The cells were harvested and stained with the apoptosis detection kit as described above, and followed by flow cytometry analysis.

### Caspase-3, Caspase-8, and Caspase-9 Activity Assays

Hela cells (6.0 × 10^5^) were seeded on six-well plates before 12 h of transfection. Cells were transfected with pEGFP-N1 or pEGFP-vp2 plasmids (500 ng per well). Forty-eight hours after transfection, cells were washed with cold PBS and prepared for the analysis of Caspase-3, -8, and -9 activities per the manufacturer’s instructions. Samples were measured at 405 nm with a microplate reader (Tecan, Sunrise) using fluorescent substrate DEVD-pNA (synthetic caspase-3 substrate), IETD-pNA (synthetic caspase-8 substrate) or LEHD-pNA (synthetic caspase-9 substrate). Data were represented as means ± standard deviations (SD) of three independent experiments. Caspase-3, caspase-8, and caspase-9 activity assay kits were obtained from BioVision.

### Yeast Two-Hybrid Screen and Colony-Lift Filter Assay

Infectious bursal disease virus *vp2* gene was subcloned into pGBKT7 plasmid to express the fusion protein GAL4-BD-VP2, used as bait and transformed into *Saccharomyces cerevisiae* AH109. Chicken spleen cDNA expression library fusion to the GAL4-activation domain codons in the pGADT7 was transformed into the *Saccharomyces cerevisiae* strain Y187. The yeast two-hybrid screen is performed following the manufacturer’s instruction (Matchmaker Two-Hybrid System 3). The selected clones were sequenced, BLASTed against the NCBI database and tested for the β-galactosidase activity. The clone transfected with pGBKT7-p53 and pGADT7-T was used as positive controls and the one transfected with pGBKT7-Lam and pGADT7-T as negative controls.

### Immunoprecipitation and Western Blot Analysis

For analysis of protein-protein interaction, Hela cells were seeded on six-well plates (6.0 × 10^5^ cells per well) and cultured for 12 h before co-transfection with pCMV-myc-*h*oraov1 and pRK5-flag-vp2, or with empty vectors as controls. Twenty-four hours after transfection, the cells were harvested and suspended in lysis buffer (50 mM Tris-HCl, pH 8.0, 150 mM NaCl, 1% NP-40, 5 mM EDTA, 10% glycerol, 10 mM dithiothreitol, 1 × completed cocktail protease inhibitor). The cell lysates were incubated with 2 μg of anti-FLAG antibody at 4°C for 2 h, followed by mixture with 20 μL beads of GammaBind^TM^ G Sepharose^TM^ (GE Healthcare) and incubation for another 4 h. Beads were washed five times with cold lysis buffer, and boiled with 20 μL 2 × SDS loading buffer for 10 min. The samples were separated on 15% SDS-polyacrylamide gels, and the resolved proteins were transferred onto PVDF membranes. Immunoblots were blocked with 5% skimmed milk, and then incubated with either anti-c-Myc or anti-FLAG antibodies. After probed with primary antibodies, the membranes were incubated with horseradish peroxidase-conjugated anti-mouse IgG antibodies. The bands were detected with an enhanced chemiluminescence (ECL) kit (Kangwei Biological Company, China).

For endogenous ORAOV1 pull-down assay, Hela cells seeded on six-well plates were transfected with pRK5-flag-vp2 or empty vector (200 ng per well). The cells were harvested 24 h post transfection and the lysates were subjected to immunoprecipitation with anti-FLAG antibody and immunoblotted with anti-ORAOV1 or anti-FLAG antibodies. To pull-down the endogenous ORAOV1 in IBDV *Lx* infected cells, DF-1 cells were mock infected or infected with IBDV *Lx* at an MOI of 10. Twenty-four hours after infection, the cell lysates were subjected to immunoprecipitation with mouse anti-VP2 McAb and immunoblotted with anti-ORAOV1 or anti-VP2 antibodies, and then followed by stripping and reprobing with rabbit anti-β-actin antibody (ab8227, Abcam).

### Confocal Laser Scanning Microscopy Assays

Hela cells (1.0 × 10^5^) were seeded on coverslips in 24-well plates and were cultured overnight before transfected with pDsRed-N1-vp2 or empty vector as controls. The one layer cells were fixed with 1% paraformaldehyde and the nuclei were stained with DAPI at 24 h after transfection. For observing the subcellular locations of endogenous protein, IBDV *Lx*- or mock- infected DF-1 cells were fixed with 1% paraformaldehyde and permeabilized with 0.2% Triton X-100 for 15 min. The cells were then blocked with 1% bovine serum albumin, and the expressions of IBDV VP2 and ORAOV1 were detected with mouse anti-VP2 monoclonal antibody and rabbit anti-ORAOV1 polyclonal antibodies, followed by TRITC-conjugated goat anti-mouse IgG and FITC-conjugated goat anti-rabbit IgG antibodies. After washes with cold PBS, the cell nuclei were stained with DAPI. For the analysis of subcellular organelle staining, cells transfected with the indicated plasmids were incubated with the MitoTracker Green in the incubator, and the procedures were performed following the manufacturer’s instructions (Beyotime). The samples were observed with a laser confocal scanning microscope (C1 standard detector; Nikon, Japan).

### Knockdown of ORAOV1 by RNAi

The siRNAs were designed by the Genechem Company (Shanghai, China) and used to knock down ORAOV1 in Hela or DF-1 cells. siRNAs targeting human *ORAOV1* (*hORAOV1*) in Hela cells included RNAi#1 (target sequence: 5′-CAA TGT CAG CCA GAC TTT- 3′), RNAi#2 (target sequence: 5′- CTT ATG ATG ACC CTA CTTA-3′), and RNAi#3 (target sequence: 5′-ATG AAG ACT TAG ACA AGAT-3′). siRNAs targeting chicken *ORAOV1* (*cORAOV1*) in DF-1 cells included RNAi#1 (target sequence: 5′-CAA GAT TGG CTC TGA GATT-3′), RNAi#2 (target sequence: 5′-CAG GTT TGT TCA ATG CTAA-3′), and RNAi#3 (target sequence: 5′-CGG ACA TGT TTG ATG AGAT-3′). Negative siRNA control (sense: 5′-UUC UCC GAA CGU GUC ACGUtt-3′), antisense: 5′-ACG UGA CAC GUU CGG AGAtt-3′). Cells (4 × 10^5^) were seeded on six-well plates and cultured for 12 h, and then were transfected with the ORAOV1 siRNA or negative siRNA using RNAi MAX reagent per the manufacturer’s instructions (Invitrogen, United States). Double transfections were performed at a 24 h interval. Cells were prepared for further analysis 24 h after the second transfection.

### RNA Isolation and Quantitative Reverse Transcription-PCR (qRT-PCR) Analysis

Total RNA was extracted from Hela or DF-1 cells using a Qiagen RNeasy kit per the manufacturer’s instructions, and treated with DNase I. One μg of total RNA was employed for cDNA synthesis by reverse transcription using an 1st Strand cDNA Synthesis kit (TaKaRa). The specific primers for human oraov1 (sense primer: 5′- TGA TGG CGG ATG AGA GGT TTC- 3′, and antisense primer: 5′- TTC CCT CCA TCA CAC CCA AAC- 3′) and human glyceraldehyde-3-phosphate dehydrogenase (GAPDH, sense primer: 5′- CAA CTA CAT GGT TTA CAT GTT CC- 3′, and antisense primer: 5′- GGA CTG TGG TCA TGA GTC CT- 3′), chicken oraov1 (sense primer: 5′- TGG CAG TTA CCT TGG ATT TG- 3′, and antisense primer: 5′- AAG TGG GGT CTT CGT ATG GG- 3′) and GAPDH (sense primer: 5′- TGC CAT CAC AGC CAC ACA GAAG- 3′, and antisense primer: 5′- ACT TTC CCC ACA GCC TTA GCAG- 3′), were designed and synthesized by Sangon Company. Real-time PCR was carried out with a Light Cycler 480 (Roche, United States). Thermal cycling parameters were as follows: 94°C for 2 min; 40 cycles of 94°C for 20 s, 56°C for 20 s, and 72°C for 20 s; and 1 cycle of 95°C for 30 s, 60°C for 30 s, and 95°C for 30 s. The final step was to obtain a melt curve for the PCR products to determine the specificity of the amplification. All samples were examined in triplicate, and the GAPDH gene was utilized as the reference gene.

### Ubiquitination Assays

For the ubiquitination assay, Hela or DF-1 cells were transfected with the indicated plasmids. Cells were treated with MG132 (10 μM) for 6 h and then lysed in 1% SDS. After boiling for 10 min, lysates were diluted 10 times with cold lysis buffer supplemented with 1× complete protease inhibitors and 10 mM *N*-ethylmaleimide (NEM). Cell lysate samples were subjected to immunoprecipitation with anti-FLAG antibody and then analyzed by Western blot.

### Measurement of IBDV Growth in DF-1 Cells

DF-1 cells were transfected with pEGFP-*c*oraov1 or pEGFP-N1. Twenty-four hours after transfection, cells were infected with IBDV at an MOI of 10. Cell cultures were harvested at different time points. The culture samples were freeze-thawed three times and centrifuged, and the viral loads in the supernatants were titrated by 50% tissue culture infective doses (TCID_50_) in DF-1 cells following the procedures as previously described (Li et al., 2012). Briefly, the samples were diluted by 10-fold in DMEM. DF-1 cells (5 × 10^4^ cells per well) were seeded in 96-well plates, and each well was added with a 100 μl aliquot of each diluted sample. Cells were cultured for 5 days at 37°C in 5% CO_2_. The cell cultural wells with a cytopathic effect (CPE) were determined to be positive.

### Statistical Analysis

The statistical comparisons were performed using GraphPad Prism software. The significance of the differences between pEGFP-vp2 transfected cells and controls in the percentage of apoptotic cells, caspase-9, -8, and -3 activities and reduction of ORAOV1, between ORAOV1 RNAi cells and controls in apoptosis, between cells with transient over expression of ORAOV1 and controls in suppression of VP2-induced apoptosis and viral growth, and between IBDV-infected cells and mock controls in the reduction of ORAOV1 was determined by the Mann-Whitney or ANOVA accordingly.

## Results

### Transient Expression of GFP-VP2 Fusion Induced Apoptosis in Hela Cells

**FIGURE 1:**
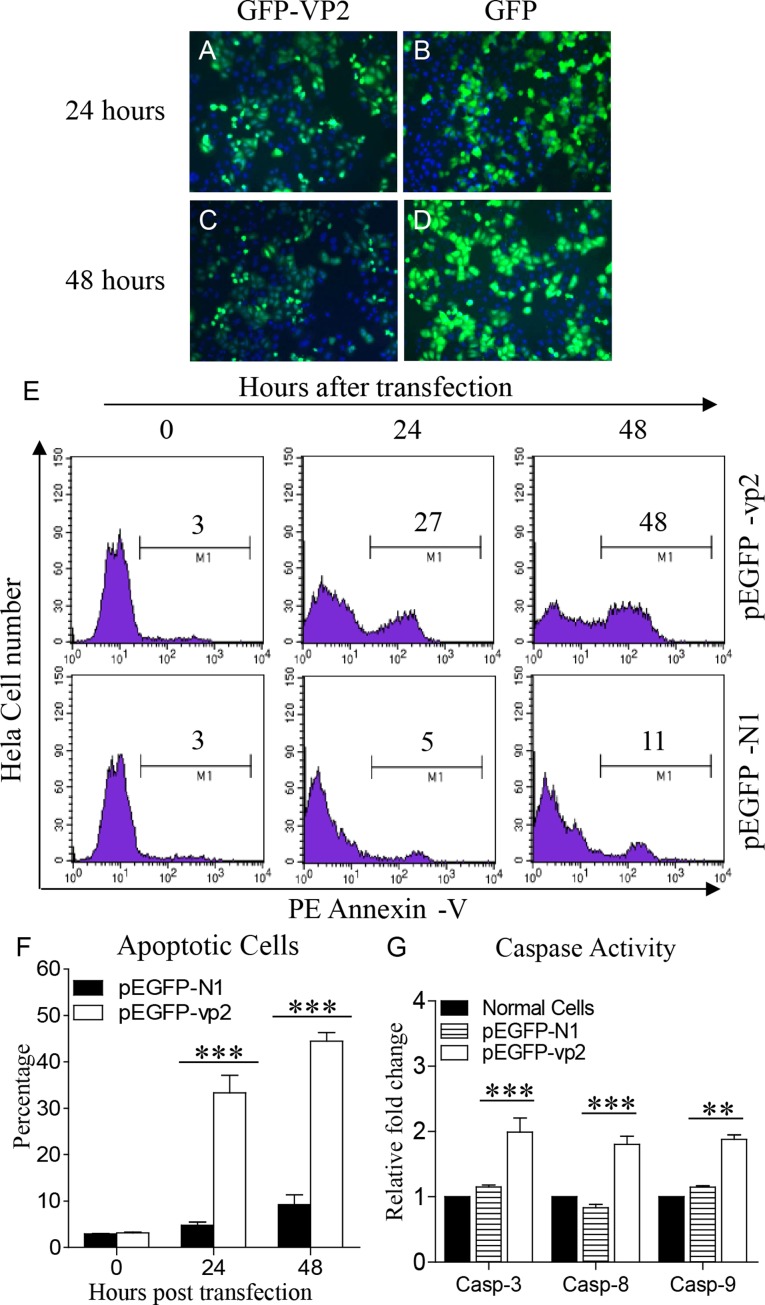
Infectious bursal disease virus (IBDV) VP2 induces apoptosis in Hela cells. **(A–D)** Expression of recombinant GFP-VP2 in Hela cells. Hela cells were transfected with pEGFP-N1-vp2 **(A,C)** or pEGFP-N1 **(B,D)** as controls. Twenty-four or 48 h after transfection, cells were stained with DAPI and examined by fluorescence microscope at indicated time points. **(E)** Transfected cells were collected and stained with PE Annexin-V. GFP-positive cells were gated for further analysis of apoptosis by flow cytometry (total cells were analyzed at 0 h). **(F)** The percentages of Annexin-V-PE positive cells. **(G)** The enzymatic activities of caspase-3, -8, and -9 in cells were examined 48 h after transfection with pEGFP-vp2. Data are representative of three independent experiments. ^∗∗∗^*p* < 0.001.

It has been demonstrated that VP2 of the IBDV Soroa strain is an apoptotic inducer in a variety of mammalian cell lines (Fernandez-Arias et al., 1997). We previously found that the expression of VP2 from another virus strain, IBDV *Lx* strain, induced apoptosis in DF-1 cells (Li et al., 2012). However, as the capsid protein with hypervariable regions, there are several differences in the amino acid sequences of VP2 between the two strains. To assess the apoptotic activity of the IBDV *Lx* VP2 in mammalian cell lines, we transfected Hela cells with pEGFP-vp2 and examined the apoptosis in these cells with flow cytometry. Both GFP-VP2 and GFP proteins were well expressed (**Figures 1A–D**). As shown in **Figures 1E,F**, transfection of Hela cells with pEGFP-vp2 induced marked apoptosis as compared to that of pEGFP-N1 transfected controls (*p* < 0.001).Consistently, the activities of caspase-3, caspase-8, and caspase-9 in pEGFP-vp2 transfected cells significantly increased as compared to that of controls (*p* < 0.001) (**Figure 1G**). These results indicate that the VP2 of IBDV *Lx* strain acts as a potent apoptosis-inducer in Hela cells.

### Interaction and Colocalization of VP2 with ORAOV1

**FIGURE 2:**
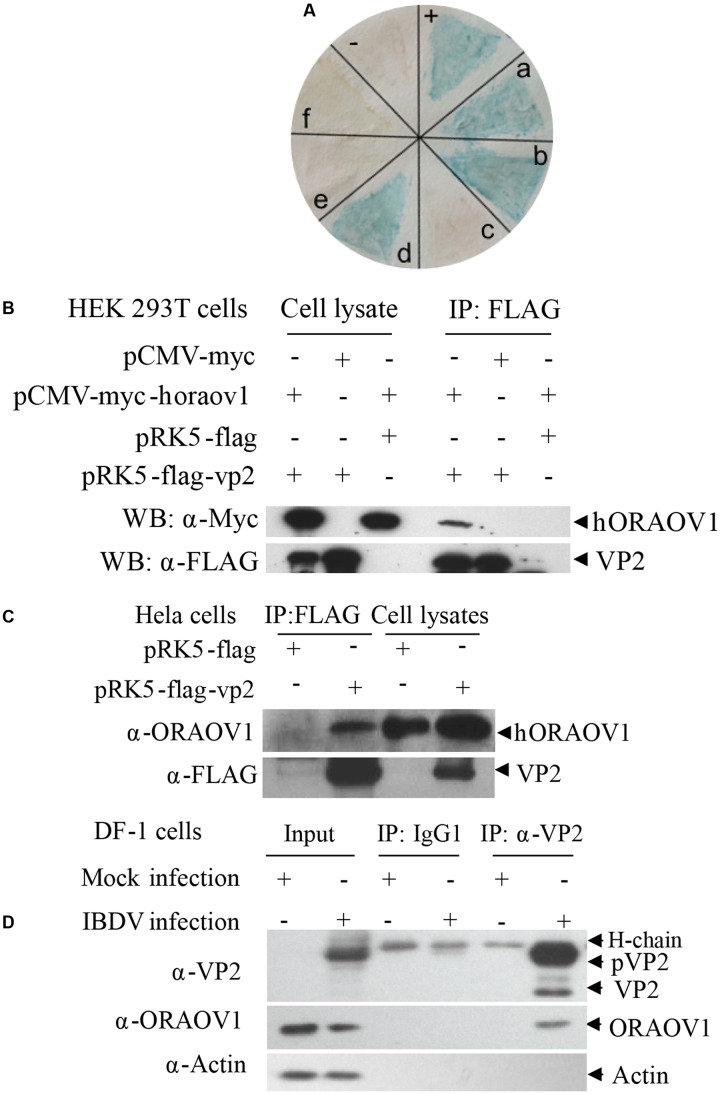
The interaction of VP2 with ORAOV1. **(A)** Colony-lift Filter assay was performed to verify the interactions of VP2 and its targets screened via yeast two-hybrid system. Negative control (–). Positive control (++). (a–f) in **(A)** represent putative clones containing pGADT7-ORAOV1, pGADT7-MICAL1, pGADT7-DDT, pGADT7-β2M, pGADT7-PCNA, pGADT7- ITGB1BP3, respectively. **(B)** The interaction of VP2 with exogenous ORAOV1. HEK293T cells were transfected with the indicated plasmids. Twenty four hours after transfection, cell lysates were prepared and co-immunoprecipitation assay was performed with anti-FLAG monoclonal antibody. The pellets were examined by Western Blot. **(C)** Interaction of VP2 with endogenous ORAOV1. Hela cells were transfected with pRK5-flag-vp2 or pRK5-flag as controls. Twelve hours after transfection, cell lysates were prepared and immunoprecipitated with anti-FLAG antibody. **(D)** Interaction of VP2 with ORAOV1 in IBDV infected cells. DF-1 cells were mock infected or infected with IBDV at an MOI of 10. Cell lysates were prepared after 24 h of infection and immunoprecipitated with anti-VP2 monoclonal antibody (IgG1) or IgG1 isotype control.

To investigate the molecular mechanism of VP2-induced apoptosis, we employed the yeast two-hybrid system using VP2 as bait to screen the chicken bursa of Fabricius cDNA library. Among the positive clones, ORAOV1 was identified 11 times, and occurred at a higher frequency among the positive clones as examined by colony-lift filter assay (**Figure 2A**), suggesting that ORAOV1 interacts with VP2. ORAOV1, a highly expressed protein in cancer (Xavier et al., 2012; Togashi et al., 2014; Li et al., 2015), plays an important role in the growth of Hela cells (Jiang et al., 2010), and in oral squamous cell carcinoma (OSCC) as a regulator of cell apoptosis (Jiang et al., 2008), which highlights its relevance to VP2 apoptotic activity. In addition, ORAOV1 gene is conserved across the eukaryotic lineage (Zhai et al., 2014). These data indicate that the apoptotic activity of VP2 might be associated with ORAOV1 functions. To determine the interaction of VP2 with ORAOV1 in cells, we subcloned human ORAOV1 into pCMV-myc vector and constructed pRK5-FLAG-vp2. We transfected HEK293T cells with these constructs and performed coimmunoprecipitation. As shown in **Figure 2B**, when the lysates of cells expressing both Flag-VP2 and Myc-ORAOV1 were immunoprecipitated with FLAG antibody, Myc-ORAOV1 was detected in the precipitate, indicating that VP2 interacted with ectopically expressed ORAOV1 in mammalian cells. Furthermore, we expressed VP2 in Hela cells and examined its interaction with endogenous ORAOV1 using pull-down assays. The binding of FLAG-VP2 with endogenous ORAOV1 was detectable in cells expressing VP2 (**Figure 2C**), indicating that VP2 interacts with endogenous ORAOV1 in host cells. To further substantiate the binding of VP2 to ORAOV1, we infected DF-1 cells with IBDV *Lx* strain and examined the interaction of VP2 with endogenous ORAOV1 using pull-down assays. Consistently, the endogenous ORAOV1 was also detected in IBDV-infected cells but not in mock infected controls (**Figure 2D**). These results clearly demonstrate that VP2 interacts with ORAOV1 in host cells.

**FIGURE 3:**
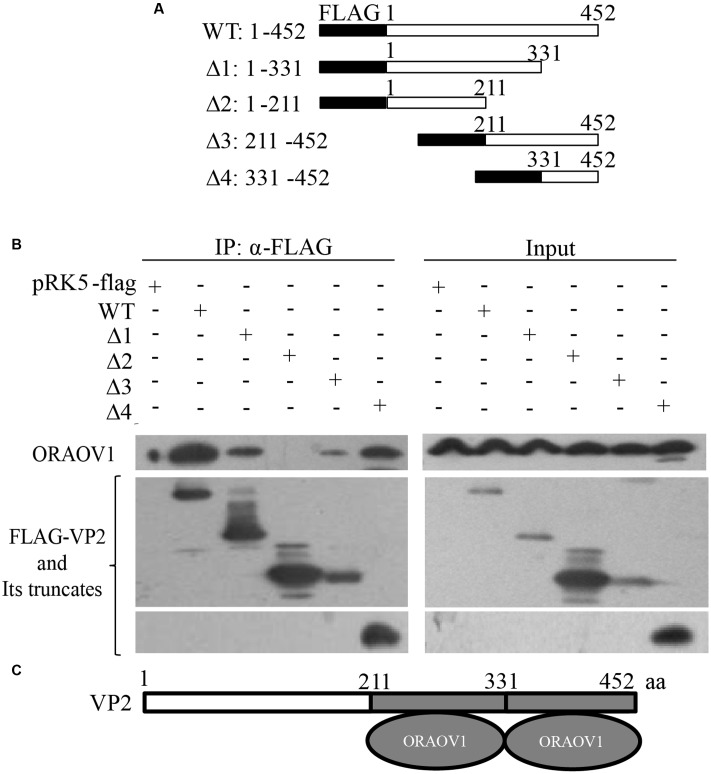
The portion of VP2 from amino acids 211–452 is responsible for binding to ORAOV1. **(A)** Schematics represent the genes encoding the full-length VP2 and truncated VP2 molecules (from Δ1 to Δ4). The numbers indicate the amino acid positions in the molecule. **(B)** Endogenous ORAOV1 interacted with different truncated VP2 mutants. HEK293T cells were transfected with full-length pRK5-flag-vp2 [wild type (WT)], the indicated truncated mutant or empty vectors. Cell lysates were prepared and immunoprecipitated with anti-Flag monoclonal antibody. The pellets were examined with Western Blot using anti-ORAOV1 and anti-FLAG antibodies. **(C)** Schematic representing the sites of VP2 binding to ORAOV1 (211–331aa and 331–452 aa).

VP2 contains a hypervariable region (HVR) responsible for the antigenic variation (Vakharia et al., 1994). Thus, we compared the amino acid sequence of IBDV *Lx* VP2 with that of published reference strains of varied virulence (Cui et al., 2013), and found that the HVR of *Lx* strain ranges from 211 to 331aa. The alignment of the HVR of *Lx* strain with that of others indicates that their sequence identity ranges from 87.6 to 100%. To rule out the possibility that the interaction of VP2 with ORAOV1 is strain-specific, we constructed a series of VP2 truncates that separate the conserved regions from the HVR based on the alignment results (**Figure 3A**), and performed immunoprecipitation. The results demonstrated that both HVR with residue of 211–331aa and conserved C-terminal domain 331–452aa (sequence similarity differing from 95.9 to 100%) retained the ability of VP2 to bind ORAOV1 (**Figures 3B,C**), indicating that the interaction of VP2 with ORAOV1 is not strain specific.

**FIGURE 4:**
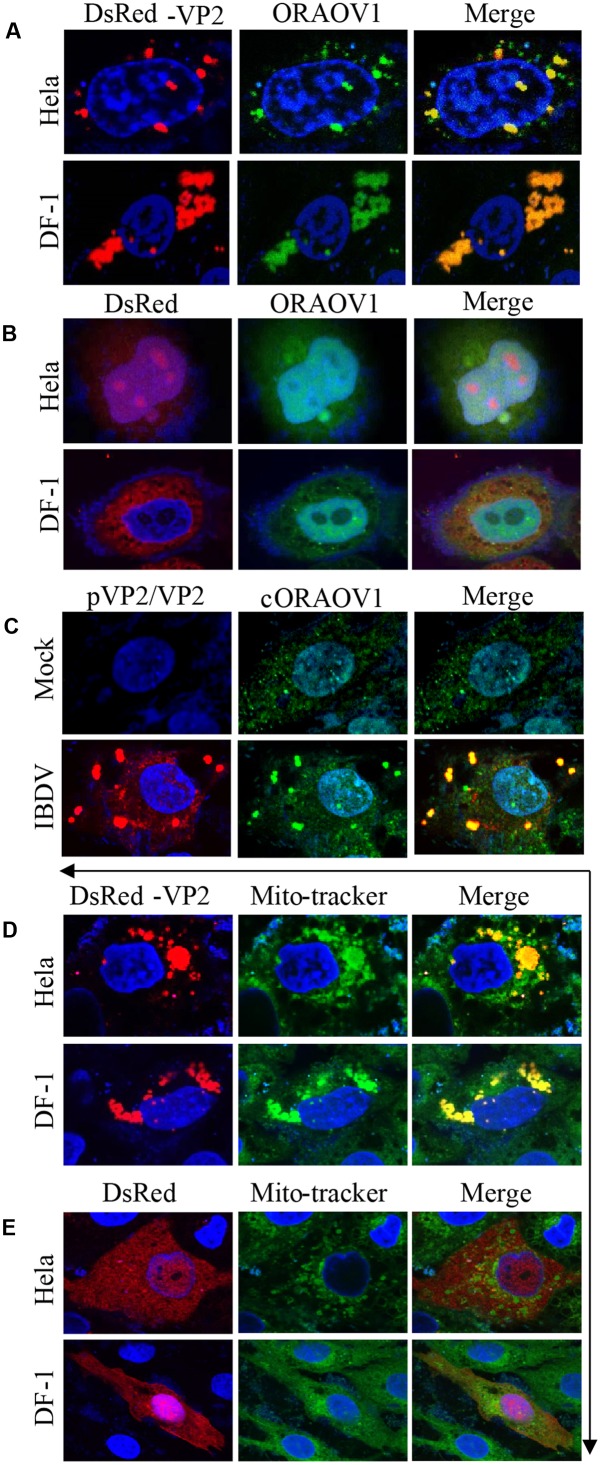
Colocalization of VP2 with ORAOV1. **(A)** Hela or DF-1 cells were seeded on 24-well plates with coverslips and transfected with pDsRed-N1-vp2. Twenty four hours post transfection, cells were fixed and immunostained with rabbit anti-ORAOV1 polyclonal antibodies, followed by incubation with FITC-conjugated goat anti-Rabbit IgG antibodies. Nuclei were counterstained with DAPI (blue). The cell samples were observed under a laser confocal scanning microscope. **(B)** DsRed control is not colocalized with ORAOV1. Hela cells or DF-1 cells were transfected with pDsRed-N1. Twenty-four hours post transfection, cells were prepared and immunostained with rabbit anti-ORAOV1, followed by FITC-conjugated anti-rabbit IgG antibodies. **(C)** DF-1 cells were mock infected or infected with IBDV at an MOI of 10. Twelve hours after infection, IBDV pVP2/VP2 and endogenous ORAOV1 were probed with mouse anti-VP2 and rabbit anti-ORAOV1 antibodies, followed by FITC-conjugated goat anti-rabbit IgG and TRITC-conjugated goat anti-mouse IgG antibodies. The samples were observed under a laser confocal scanning microscope. **(D,E)** Localization of VP2 in the mitochondria. Both Hela and DF-1 cells were transfected with pDsRed-VP2 **(D)** or pDsRed-N1 **(E)** as controls. Twenty-four hours after transfection, cells were stained by MitoTracker Green for the mitochondrion. The cell samples were observed under a laser confocal scanning microscope.

To unveil the subcellular localizations of VP2 and endogenous ORAOV1, we performed confocal microscopy assay with Hela and DF-1 cells, which transiently expressed DsRed-VP2 and were probed with rabbit anti-ORAOV1 polyclonal antibodies, followed by staining with FITC-conjugated goat anti-rabbit antibodies. The results show that DsRed-VP2 and endogenous ORAOV1 proteins colocalized in the cytoplasm of pDsRed-vp2 transfected cells (**Figure 4A**). In contrast, DsRed control was not colocalized with ORAOV1 (**Figure 4B**). Furthermore we infected DF-1 cells with IBDV at an MOI of 10 and examined the colocalization of viral VP2 and ORAOV1. Since the anti-VP2 monoclonal antibody employed in the present study recognizes 394 to 410aa, the positive structures detected by the antibody included pVP2 and VP2. Endogenous ORAOV1 was evenly distributed in DF-1 cells with mock infection (**Figure 4C**). However, in IBDV infected cells, ORAOV1 was mostly distributed in the form of granular structures and colocalized with accumulated pVP2/VP2. These results strongly support the findings that pVP2/VP2 interacts with ORAOV1 in cells. Furthermore, we found that the accumulated VP2 were primarily located in the mitochondria of Hela and DF-1 cells (**Figures 4D,E**).

### Knockdown of ORAOV1 by RNAi Induced Apoptosis

**FIGURE 5:**
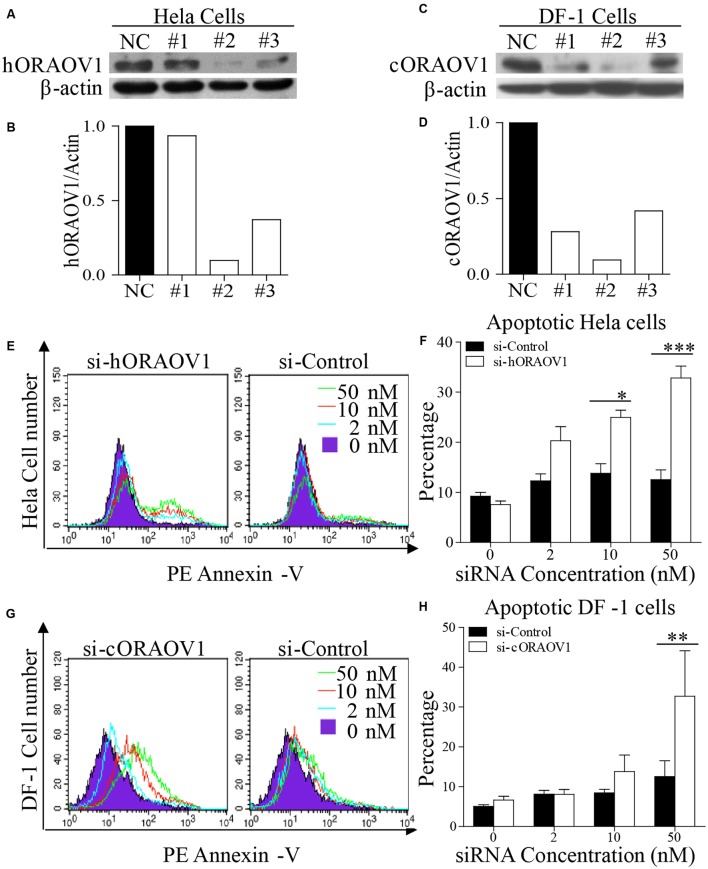
Knockdown of ORAOV1 induces apoptosis in a dose-dependent manner. **(A–D)** Effects of different RNAi constructs on endogenous ORAOV1 expression. **(A)** Hela cells were transfected with siRNA (RNAi#1–3) or controls targeting human ORAOV1. Forty-eight hours after the second transfection, cell lysates were prepared and examined with Western Blot using anti-ORAOV1 antibody. β-actin expression was used as an internal control. **(B)** The relative levels of human ORAOV1 in ORAOV1 RNAi-treated Hela cells. The density of protein bands in panel A was quantitated by densitometry, and normalized with the density of the β-actin bands. **(C)** As in **(A)**, DF-1 cells were transfected with siRNA (RNAi#1-3) or controls targeting chicken ORAOV1. **(D)** The relative levels of chicken ORAOV1 expression in the RNAi-treated DF-1 cells. **(E–H)** Knockdown of ORAOV1 by RNAi induces apoptosis in a dose-dependent manner. RNAi construct#2 in **(A)** or **(C)** were used to knockdown ORAOV1 in Hela or DF-1 cells. Forty-eight hours after the second transfection, Hela or DF-1 cells were harvested and stained with PE annexin-V, followed by flow cytometry analysis **(E,G)**. Percentages of the annexin-V positive cells in the indicated groups were calculated, and the significance of the difference between ORAOV1-RNAi and control-RNAi treatments was performed by ANOVA **(F,H)**. Data are representative of three independent experiments. ^∗^*p* < 0.05, ^∗∗^*p* < 0.01, and ^∗∗∗^*p* < 0.001.

ORAOV1 is commonly overexpressed in solid tumors. It has been reported that knockdown of ORAOV1 results in tumor cell apoptosis (Jiang et al., 2008, 2010). As the amino acid sequence similarity of human and chicken ORAOV1 is 66.4%, we examined the effect of silencing human and chicken ORAOV1 on apoptosis. We made three ORAOV1 RNAi constructs, and found that the constructs could effectively lower the cellular level of *h*ORAOV1 and cORAOV1 in Hela cells and DF-1 cells, respectively, without causing discernable changes in cell morphology (**Figures 5A–D**). As expected, when examined by flow cytometry using Annexin-V staining, cells receiving the siRNA against *h*ORAOV1 or *c*ORAOV1 displayed marked apoptotic changes (**Figures 5E–H**), while transfection with siRNA-resistant form of ORAOV1 in oraov1 RNAi-treated Hela cells rescued the ORAOV1 knockdown effect (Supplementary Figure S1). These data indicate that ORAOV1 plays a critical role as an antiapoptotic protein in both human and avian cells.

### Reduction of ORAOV1 by Expression of VP2 or IBDV Infection

**FIGURE 6:**
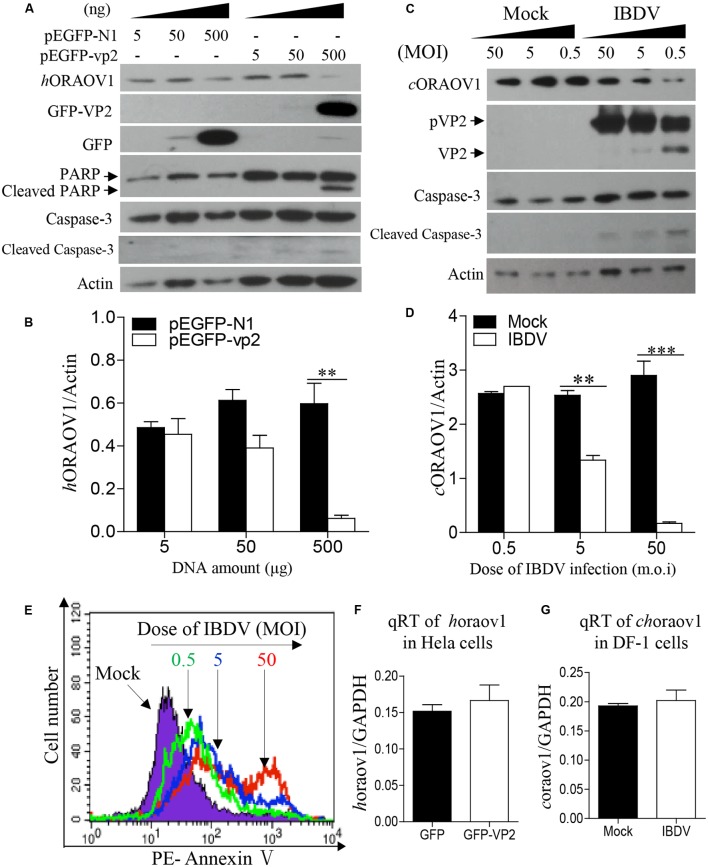
VP2 reduces ORAOV1. **(A–D)** The ORAOV1 expression is reduced by VP2. Hela cells in 6-well pate were transfected with the indicated amounts of pEGFP-N1-vp2 or pEGFP-N1 controls. Total amounts of plasmid DNA were equalized to 500 ng using pRK5-flag. Forty eight hours after transfection, cell lysates were examined with Western Blot **(A)**. The densities of bands in A were quantitated by densitometry. The relative levels of ORAOV1 were calculated as follows: band density of ORAOV1/ that of β-actin **(B)**. **(C)** DF-1 cells were mock infected or infected with IBDV at different MOI of 0.5, 5, or 50. Mock infected cells were treated in the same way as IBDV infection at the indicated MOI but using culture medium instead of the virus 24 h after infection, the lysates of IBDV-infected cells were examined with Western Blot. The densities of bands in **(C)** were quantitated by densitometry as described above **(D)**. Data are representative of three independent experiments, ^∗∗^*p* < 0.01, ^∗∗∗^*p* < 0.001. **(E)** The reduction of ORAOV1 correlated to the enhanced apoptosis during IBDV infection. Cell samples in **(C)** were collected and stained with PE annexin-V for apoptosis analysis by flow cytometry. **(F)** Examination of oraov1 mRNA levels in VP2 transfected cells. Hela cells were transfected with the 500 ng pEGFP-N1-vp2 or pEGFP-N1 controls. The cell samples were collected for qRT-PCR assay 48 h post transfection. **(G)** Examination of oraov1 mRNA levels in IBDV infected cells. DF-1 cells were mock infected or infected with IBDV at an MOI of 5. Twenty four hours after infection, the cell samples were collected for qRT-PCR assay. The expression levels of mRNA were calculated in relation to that of GAPDH. Results are representative of three independent experiments.

**FIGURE 7:**
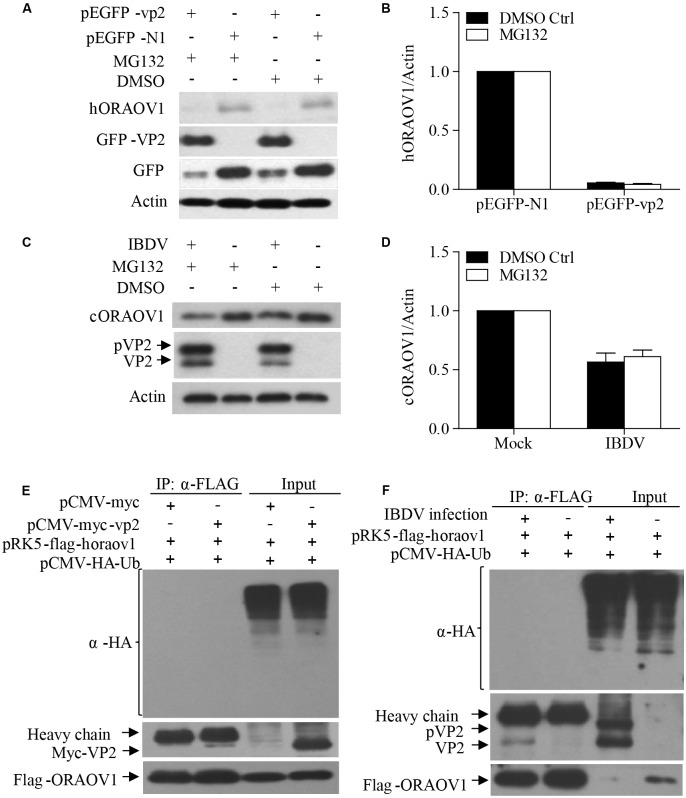
The reduction of ORAOV1 by VP2 does not involve the proteasome protein degradation pathway. **(A–D)** The reduction of ORAOV1 VP2 in Hela cells was not affected by MG132. **(A)** Hela cells were transfected with the indicated plasmids. Forty-eight hours post transfection, cells were treated with MG132 or DMSO for 6 h, and followed by examination with Western Blot. **(B)** The ratio of hORAOV1 protein density over that of actin in pEGFP-N1 transfected cells or mock infected controls was normalized to 1.0 and presented on the bar graph. DF-1 cells were transfected with the indicated plasmids. Forty-eight hours post transfection, cells were infected with IBDV at an MOI of 10. **(C)** Twenty-four hours post infection, cells were treated with MG132 or DMSO for 6 h, and followed by examination with Western Blot. **(D)** The ratio of cORAOV1protein density over that of actin in mock or IBDV infected cells was normalized to 1.0 and presented on the bar graph. β-actin expression was used as an internal control. Data are representative of three independent experiments. **(E,F)** VP2 did not affect ORAOV1 ubiquitination. Hela cells were transfected with the indicated plasmids and 24 h post transfection, cells were treated with MG132. Total cell lysates were prepared and immunoprecipitated with α-FLAG antibody, immunoblotted with anti-HA, anti-Myc or anti-Flag antibodies **(E)**. HEK293T cells were transfected with pCMV-HA-Ub. Twenty-four hours post transfection, cells were mock or IBDV infected at an MOI of 10 for 24 h, and followed by treatment with MG132. Total cell lysates were prepared and immunoprecipitated with α-FLAG antibody, immunoblotted with anti-HA, anti-vp2 or anti-Flag antibodies **(F)**.

The facts that knockdown of ORAOV1 induces apoptosis, and VP2 interacts with ORAOV1 and induces apoptosis, suggest that expression of VP2 by transfection or IBDV infection would therefore reduce ORAOV1 in cells. To test this hypothesis, we examined the ORAOV1 levels in Hela cells with pEGFP-vp2 transfection or DF-1 cells with IBDV infection. As shown in **Figures 6A,B**, transfection of cells with pEGFP-vp2 at a dose of 500 μg or over significantly reduced *h*ORAOV1 in cells (*p* < 0.01), accompanied by the cleavages of Caspase-3 and its substrate PARP. Importantly, the ORAOV1 level was also markedly reduced in IBDV-infected DF-1 cells (**Figures 6C,D**), accompanied with Caspase-3 clevage and the increased rate of apoptotic cells (**Figure 6E**). Notably, the mRNA expressions of oraov1 in pEGFG-vp2 transfected or IBDV infected cells have no significant changes as compared to that of controls (**Figures 6F,G**). To determine whether the expression of VP2 in pRK5-flag-vp2 transfected cells was commensurate with viral VP2 expression in IBDV-infected cells, we examined VP2 expressions in pRK5-vp2 transfected DF-1 cells with that of IBDV-infected ones using Western Blot. The expression of VP2 in pRK5-flag-vp2 transfected DF-1 cells was comparable with that of VP2 in IBDV-infected cells (data not shown), indicating that transfection with pRK5-flag-vp2 could mimick IBDV infection in terms of VP2 expression and can be used to investigate the biological activities of VP2 in host cells. The findings that VP2 reduces ORAOV1 in cells prompted us to investigate the mechanism underlying VP2-induced reduction of ORAOV1. As ubiquitin-proteasome pathway plays a central role in protein degradation, we examined the inhibitory effect of MG132, a proteasome inhibitor, on ORAOV1 reduction in pEGFP-vp2 transfected Hela cells and IBDV-infected DF-1 cells. The result showed that MG132 could not block VP2- or IBDV-induced reduction of ORAOV1 (**Figures 7A–D**). Furthermore, we examined the ubiquitination of FLAG-ORAOV1 in cells transfected with HA-Ub expression constructs in the presence of VP2. Neither Myc-VP2 nor viral VP2 of IBDV caused ORAOV1 ubiquitination (**Figures 7E,F**), indicating that the reduction of ORAOV1 by VP2 expression or IBDV infection is irrelevant to proteasome-mediated protein degradation pathway.

### The C-terminal of VP2 (331–452aa) Triggered ORAOV1 Degradation and Apoptosis

**FIGURE 8:**
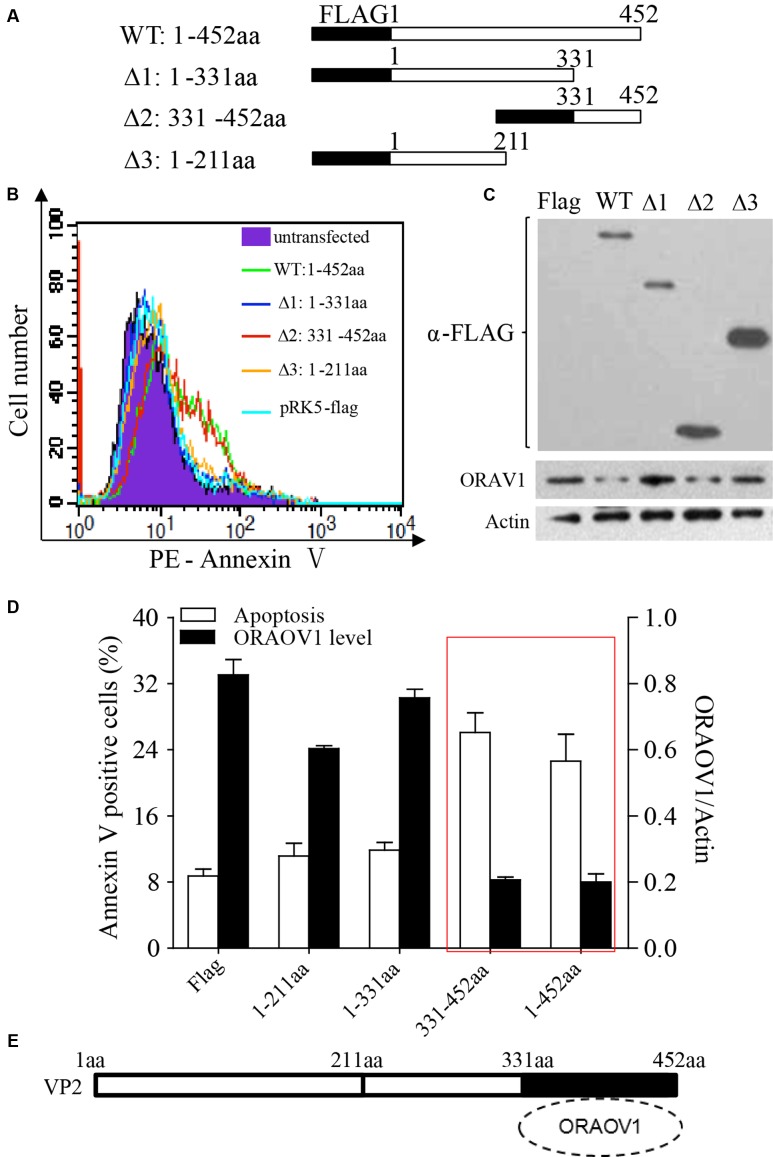
The C-terminal of VP2 (331–452aa) is essential to promote the degradation of ORAOV1 and cell apoptosis. **(A)** Schematics represent the genes encoding the full-length VP2 and truncated VP2 molecules (from Δ1 to Δ3). The numbers indicate the amino acid positions in the molecule. **(B)** Hela cells were transfected with full-length pRK5-flag-vp2 (WT), the indicated truncated vp2 or empty vectors. After 48 h of transfection, total cells were stained with Annexin PE annexin-V and examined by flow cytometry for apoptosis. **(C)** Cell samples in B were examined by Western Blot. **(D)** Percentages of the annexin-V positive cells and the ORAOV1 expression levels in **(B)**. **(E)** The portion of VP2 from amino acids 331–452 is responsible for ORAOV1 degradation and VP2-induced apoptosis.

To determine the region of VP2 that is responsible for the reduction of ORAOV1 and the consequent cell apoptosis, we transfected Hela cells with different lengths of truncated VP2 and examined the apoptosis and the expression of OROAV1 in these cells. As shown in **Figure 8**, Like the full-length of VP2, the conserved C-terminal domain 331–452aa (Δ2) significantly induced cell apoptosis and ORAOV1 reduction, while other truncated VP2 had no effects on both cell viablity and ORAOV1 expression. These data indicate that the C-terminal of VP2 (331–452aa) may act as a functional domain to induce ORAOV1 reduction and cell apoptosis.

### Inhibition of VP2-Induced Apoptosis by Overexpression of ORAOV1

**FIGURE 9:**
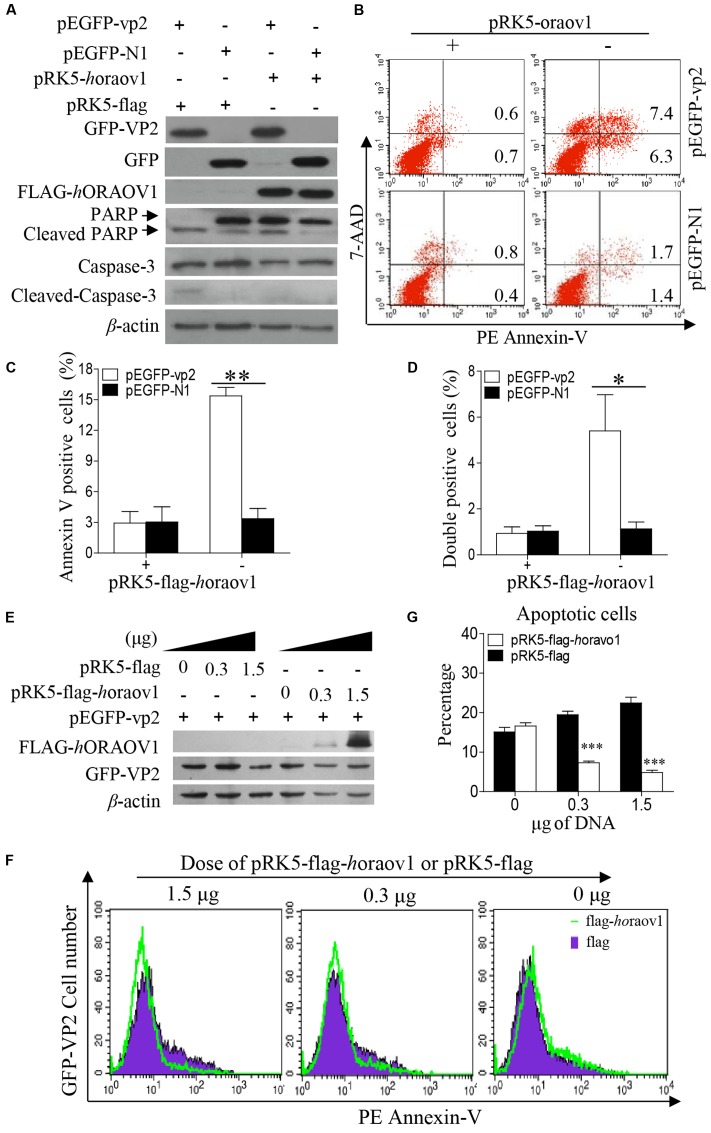
Overexpression of ORAOV1 suppressed VP2-induced apoptosis. **(A)** Hela cells in 6-well plate were transfected with 0.3 μg of pEGFP-N1-VP2 or pEGFP-N1 as controls, together with 1.5 μg of pRK5-flag-horaov1 or the same amount of pRK5-flag. **(B)** Cells treated as in **(A)** were stained with PE annexin-V, and GFP-positive cells were examined with flow cytometry for apoptosis. **(C,D)** The percentage of apoptotic cells expressing GFP-VP2 or GFP in the presence of overexpressed ORAOV1. Data are representative of three independent experiments. **(E)** Hela cells were transfected with 300 ng of pEGFP-vp2 together with indicated doses of pRK5-flag-horaov1 or pRK5-flag. The total amount of plasmid DNA were equalized to 2 μg using an irrelevant vector pcDNA-4.0. **(F,G)** Cells treated as in **(E)** were stained with PE annexin-V, and GFP-positive cells were examined with flow cytometry for apoptosis. The percentages of VP2 induced apoptotic cells were reduced by the ORAOV1 expression in a dose-dependent manner. Data are representative of three independent experiments. ^∗^*p* < 0.05, ^∗∗^*p* < 0.01, ^∗∗∗^*p* < 0.001.

Since VP2 induced apoptosis accompanied with reduction of ORAOV1, it was very likely that enhancement of ORAOV1 expression would inhibit VP2-induced apoptosis. Thus, we transfected Hela cells with pRK5-flag-horaov1 or pRK5-flag controls along with pEGFP-vp2 or pEGFP-N1 controls, and examined the apoptosis of transfected cells with flow cytometry. We found that the cells expressing FLAG-*h*ORAOV1 fusion markedly suppressed VP2-induced apoptosis and the cleavages of Caspase-3 and PARP (**Figures 9A–D**). Furthermore, we transfected cells with different doses of pRK5-flag-ORAOV1 together with pEGFP-vp2 and examined the apoptotic cells (**Figures 9E–G**). VP2-induced apoptosis was markedly inhibited by overexpressed *h*ORAOV1 in Hela cells. These results strongly support the above findings that VP2 induces apoptosis via reduction of ORAOV1 in cells, indicating that the ORAOV1 plays a crucial role in VP2-induced apoptosis.

### Overexpression of ORAOV1 Suppresses IBDV Induced Apoptosis and Restricts the Viral Release Early after Infection

**FIGURE 10:**
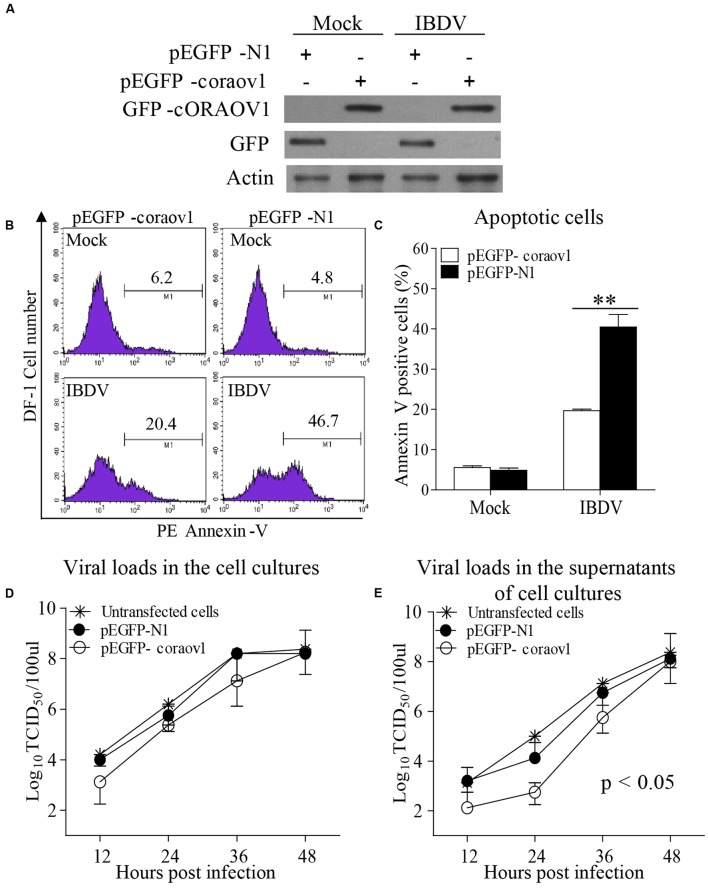
ORAOV1 inhibited IBDV induced apoptosis accompanied by restriction of viral release early after infection. **(A)** DF-1 cells were transfected with pEGFP-horaov1 or pEGFP-N1 as controls. Twenty four hours after transfection, cells were mock infected or infected with IBDV at an MOI of 10. The expressions of GFP-cORAOV1, GFP and actin controls in DF-1 cells were examined with Western Blot using corresponding antibodies. **(B)** The cell samples in **(A)** were stained with PE annexin-V, and GFP-positive cells were examined with flow cytometry for apoptosis. **(C)** Percentages of IBDV-induced apoptotic cells with overexpression of cORAOV1. Data are representative of three independent experiments, and statistically analyzed with ANOVA. ^∗∗^*p* < 0.01. **(D,E)** Effects of ORAOV1 expression on IBDV replication. DF-1 cells expressing GFP or GFP-cORAOV1 were infected with IBDV at an MOI of 10. The viral loads in the cell cultures **(D)** and supernatants **(E)** at the indicated time points after infection were determined by TCID_50_. The significance of the difference between the cells expressing GFP-cORAOV1 and the control groups was performed by ANOVA (*p* < 0.05). The graph shows the average of viral titers in DF-1 cells from three individual experiments.

To consolidate the above findings, we examined the effect of expressing GFP-cORAOV1 fusion on IBDV-induced apoptosis. We transfected DF-1 cells with pEGFP-*c*oraov1 or pEGFP-N1 as controls followed by infection with IBDV at an MOI of 10. Twenty-four hours after IBDV infection, cells were collected for the measurement of ORAOV1 expression and apoptosis. The results show that expression of GFP-ORAOV1 significantly suppressed apoptosis in cells with IBDV infection (**Figures 10A–C**) (*p* < 0.01), indicating that ORAOV1 is involved in IBDV-induced apoptosis.

Apoptosis is a defense mechanism of host cells in response to virus infection in order to limit viral propagation. It was found that IBDV-induced apoptosis affected the virus growth (Yao and Vakharia, 2001; Li et al., 2012). Thus, we attempted to determine whether overexpression of *c*ORAOV1 affects the replication of IBDV in DF-1 cells. We then transfeceted DF-1 cells with pEGFP-*c*oraov1 and examined the viral replication in these cells by measuring viral loads in the culture of IBDV infected cells at different time points post IBDV infection. We found that cORAOV1 overexpression markedly restricted IBDV release in the supernatants of the cell cultures compared to that of controls early post infection (**Figure 10E**) (within 24 h, *p* < 0.05), whereas the viral loads in the total cell cultures or the viral release at later stage were unaffected (**Figure 10D**). These results suggest that IBDV take advantage of VP2-induced apoptosis via reduction of ORAOV1 to facilitate viral spread at an early stage of viral infection.

## Discussion

It is well established that IBDV infection induces apoptosis in target cells of the bursa of Fabricius, triggering immunosupression (Vasconcelos and Lam, 1994, 1995; Tham and Moon, 1996; Shahsavandi et al., 2014; Liang et al., 2015). Transient expression of IBDV *vp2* gene containing the first 452 codons led to cell apoptosis and such activities were found not only in host cells but also in a variety of mammalian cell lines (Fernandez-Arias et al., 1997). Furthermore, VP2 with 452 residues was detectable during IBDV infection (Irigoyen et al., 2012). Thus, we focused on the mechanism of VP2 (452 residues) induced cell apoptosis. Consistently, we found that the VP2 of *Lx*, a IBDV strain adapted to growth in DF-1 cells, induced marked cell apoptosis with the activation of caspase-3, caspase-8, and caspase-9 in Hela cells (**Figures 1E–G**) as well as in DF-1 cells as previously decribed (Li et al., 2012). In our attempts to search for the particular cellular components interacting with VP2 and associated with the cell apoptosis, ORAOV1, a cellular protein commonly overexpressed in solid tumor cells (Jiang et al., 2008), shows its relevance to VP2 induced apoptosis. ORAOV1, originally identified as an amplification-dependent candidate oncogene of human tumor, is conserved at chromosome band 11q13, one of the most prevalent amplified regions associated with human cancer (Huang et al., 2002). It was found that knockdown of ORAOV1 induces S-phase cell cycle arrest followed by cell apoptosis and cleavages of Caspase-3, Caspase-8, and Caspase-9 (Jiang et al., 2010). However, the apoptotic response is independent of the cells with amplified ORAOV1 because such phenotype was also observed in the cancer cell lines without the up-regulation of ORAOV1, including Tca8113 and CAL-27 (Jiang et al., 2008). It implies that a basal level of ORAOV1 is sufficient to maintain tumor cell viability. We found that Hela cells expressing VP2 shared a common fate with the same cell line lacking ORAOV1. This aroused our interest in probing into the role of ORAOV1 in VP2-induced apoptosis.

Frist, we found that knockdown of ORAOV1 by RNAi induced apoptosis, supporting the previous finding of ORAOV1 as an anti-apoptotic molecule (Jiang et al., 2010). Secondly, we found that the level of cellular ORAOV1 remarkably decreased in the pEGFP-vp2 transfected or IBDV infected cells with no change of the mRNA expression, and the ORAOV1 reduction highly correlated with VP2 expression and VP2-induced apoptosis. These results account for the difficulties of identifying VP2-ORAOV1 interaction at the late stage of IBDV infection or VP2 transfection, in which a large number of cells undergo apoptosis. Thus, when we performed the pull-down or confocal microscopy assays, it was important to harvest the cell samples early post IBDV infection at a lower MOI and transfect cells with VP2 plasmids at a half dose to avoid over reduction of ORAOV1 by VP2. In contrast, overexpression of ORAOV1 suppressed VP2-induced apoptosis. These facts indicate that VP2 reduced ORAOV1, an antiapoptotic cellular protein, leading to apoptosis. Therefore, it is of importance to find out the interplay between VP2-ORAOV1 binding and the decreased ORAOV1. Based on our findings, ORAOV1 was not ubiquitinated in cells with VP2 expression or IBDV infection, and reduction of ORAOV1 by VP2 was not affected by MG132, an inhibitor of proteasomal protein degradation. These findings indicate that ORAOV1 degradation is independent of the Proteasome-Ubiquitin pathway.

It has been proved that the C-terminal domain of pVP2 (441–512 aa) is essential for the interaction between VP2 and VP3 (Ona et al., 2004), and the co-expression of VP3 can counteract the VP2 (452 residues) induced apoptosis (Busnadiego et al., 2012). Here we show that the C-terminal of VP2 (331–452aa), a binding site of VP2 for ORAOV1 that partially covers the binding site of VP2 to VP3, is essential to promote the ORAOV1 reduction and cell apoptosis. Therefore, we postulate that the interaction of VP2 to ORAOV1 is competitively blocked by VP3, which impairs ORAOV1 degradation induced apoptosis. Several questions need to be addressed. For example: Is VP2 a protease? and is ORAOV1 a substrate for VP2? It has been established that pVP2 has autoproteolytic activity and the mutant D431N can abolish such endopeptidase activity (Irigoyen et al., 2009). Since the catalytic site of endopeptidase activity is in the C-terminal of VP2 (331–452aa), we then investigated the effect of the proteolytic activity in Asp431 of VP2 on ORAOV1 degradation. However, the transfection of pEGFP-vp2-D431N was still able to induce apoptosis as well as the degradation of ORAOV1 in Hela cells or DF-1 cells (Data not shown). Thus, the endopeptidase activity of VP2 Asp431 is irrelevant to the apoptosis. The mechanism of ORAOV1 reduction by VP2 still remains elusive. More efforts will be required to elucidate the mechanism of VP2-induced apoptosis via ORAOV1.

As an important regulator in the cellular antioxidant system, ORAOV1 in ESCC cells protects the tumor cells from ROS-induced damage by binding to pyrroline-5-carboxylate reductase (PYCR) (Togashi et al., 2014), which can directly scavenge ROS and upregulate the level of antioxidant enzymes (Krishnan et al., 2008). In parallel, ORAOV1 is found in a complex with ATP-binding cassette (ABC)-ATPase (ABCE1) which can alleviate the ribosomal damage generated by ROS (Zhai et al., 2014). The loss of ORAOV1 function could lead to ROS imbalance, resulting in programmed cell death. This may explain our finding that VP2 induces apoptosis via reduction of ORAOV1 and the published data by others that VP2 activates the double-stranded RNA (dsRNA)-dependent protein kinase (PKR) and then leads to the phosphorylation of the eukaryotic initiation factor 2a (eIF2a), initiating an apoptotic response (Busnadiego et al., 2012). Several reports have related the oxidative stress to PKR pathway activation (Wang et al., 2007; Pyo et al., 2008; Li et al., 2010). Interestingly, an increased ROS level is observed in IBDV infected host cells (Zhang et al., 2011). Therefore,, it is very likely that the reduction of ORAOV1 by VP2 results in the impairment of ROS which in turn activates the PKR-dependent apoptosis. On the other hand, stable overepxpression of ORAOV1 was reported to enhance cell growth and the resistance to oxidative stress treatment in the KYSE70 and KYSE170 cell lines, which belong to esophageal squamous cancer cells (ESCC) (Togashi et al., 2014). We also found that overexpression of ORAOV1 significantly suppressed VP2-induced apoptosis in Hela cells. However, overexpression of cORAOV1 in the DF-1 cells with IBDV infection partially suppressed IBDV-induced apoptosis, which agrees with our previous finding that IBDV VP2 is one of the two apoptotic inducer in host cells, and the other is VP5 (Li et al., 2012; Lin et al., 2015). In addition, DF-1 cells with overexpression of cORAOV1 restricted viral release early after IBDV infection, whereas there was no significant changes of the virus replication or the viral release at a later stage. It seems that IBDV might take advantage of VP2-induced apoptosis via degrading ORAOV1 to facilitate its release. Furthermore, ORAOV1 has recently been characterized as an adaptor which recruits ABCE1 to the cytosolic iron-sulfur (Fe-S) protein assembly (CIA) machinery for Fe-S cluster insertion, which is indispensible for the maturation of functional ABCE1 (Desiree et al., 2015). ABCE1 is reported to directly influence protein synthesis in cells, involving the nuclear export of ribosomal subunits (Hopfner, 2012), and belongs to the most conserved proteins in Eukarya and Archaea (Becker et al., 2012). The depletion of ORAOV1 impairs Fe-S cluster formation on ABCE1 and consequently leads to protein translation arrest (Desiree et al., 2015), which fits into the published data that VP2 inhibits total protein synthesis in different cell lines (Fernandez-Arias et al., 1997). Thus, it is likely that the degradation of ORAOV1 by VP2 triggers the blockage of protein synthesis via impeding the Fe-S cluster assembly on ABCE1, resulting in apoptosis. Moreover, ABCE1 is imported from the cytoplasm into the mitochondria (Le Roy et al., 2001). Based on the finding that ORAOV1 is the crucial adaptor of ABCE1 (Desiree et al., 2015) and it predominantly binds to accumlated VP2 which is colocalized with mitochondrion, we proposed that VP2 might interact with ORAOV1 in the mitochondria. Our data show that the high level of MitoTracker positive structures displayed in DF-1 cells (**Figures 4D,E**), which is in agreement with the previous report (Kim et al., 2001). In addition, Caspase-9, an apoptotic signal that is linked to mitochondria-mediated apoptosis, was activated in VP2-transfected cells in the present study, and Bcl-2, one of the key regulators of the permeabilization of the outer mitochondria membrance (Wu and Bratton, 2013), inhibited VP2-induced apoptosis (Fernandez-Arias et al., 1997). Thus, it is possible that VP2 lead to extensive production of ROS in stressed mitochondira by trigging ORAOV1 degradation, and the accumulated ROS could enhance the release of cytochrome c (Ott et al., 2002) and activate the mitochondria-mediated apoptosis. However, the activation of caspase-8 by VP2 implies that VP2 induced cell death is not dependent on mitochondria-mediated apoptosis only. Since the knockdown of ORAOV1 can cause Caspase-8 cleavage (Jiang et al., 2010), ORAOV1 degradation may be also responsible for VP2-triggered Caspase-8 activation.

In summary, our data provide evidence that VP2 interacts with ORAOV1 and induces apoptosis via reduction of ORAOV1. In contrast, overexpression of ORAOV1 inhibits VP2-induced apoptosis, indcating that ORAOV1 plays an antiapoptotic role in VP2-induced apoptosis. Thus, the reduction of ORAOV1 by VP2 may account for VP2-induced apoptosis in various cell lines. As the role of ORAOV1 in the pathogenesis of infectious agents has not been reported, this study uncovers a potentially important pathway of how pathogens infect host by interacting with ORAOV1. Further investigations into the molecular mechanisms of VP2-induced ORAOV1 reduction and apoptosis will help to elucidate the pathogenesis of viral infection, and shed lights on the development of VP2 recombinant DNA vaccine against IBDV, along with the development of cancer therapy with VP2 of apoptotic effect.

## Author Contributions

SZ and YQ designed and conceived the experiments, conducted data analysis and wrote the manuscript. YQ performed the experiments. ZX and YW analyzed the data and guided experimental design for cell apoptosis study. XL and HC contributed reagents/materials/analysis tools.

## Conflict of Interest Statement

The authors declare that the research was conducted in the absence of any commercial or financial relationships that could be construed as a potential conflict of interest.
