# Supplementary figures and images for: VP2 of Infectious Bursal Disease Virus Induces Apoptosis via Triggering Oral Cancer Overexpressed 1 (ORAOV1) Protein Degradation
Source: Front Microbiol. 2017 Jul 19;8:1351. doi: 10.3389/fmicb.2017.01351 (PMC5515827; doi:10.3389/fmicb.2017.01351)

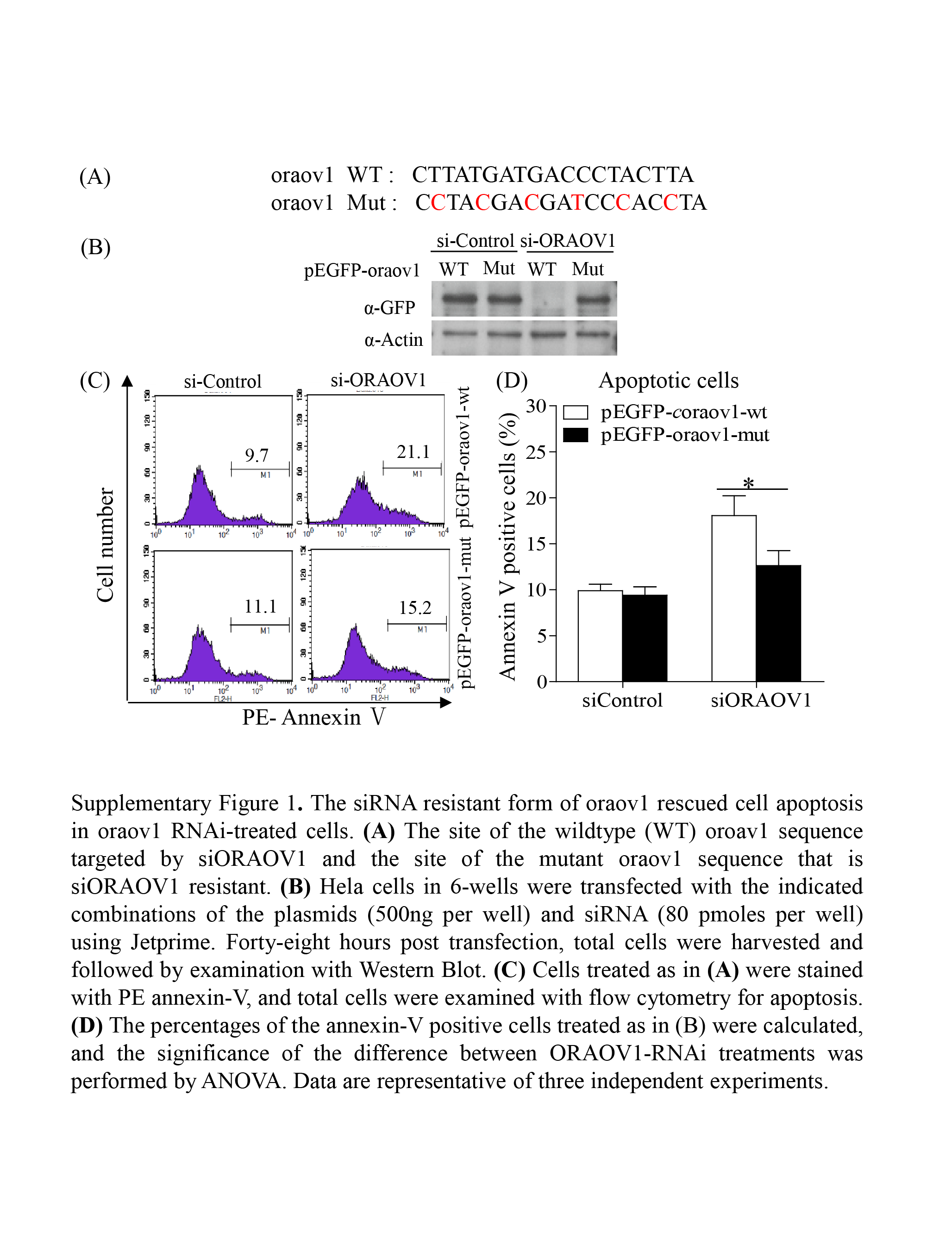

Supplement: Supplementary file 1 [file Image_1.TIF]
